# Exploring the contribution of self-help groups to sexual and reproductive health and HIV outcomes for female sex workers in sub-Saharan Africa: A scoping review

**DOI:** 10.1371/journal.pgph.0003883

**Published:** 2025-04-24

**Authors:** Gracious Madimutsa, Fortunate Machingura, Owen Nyamwanza, Frances M. Cowan, Webster Mavhu

**Affiliations:** 1 Centre for Sexual Health and HIV/AIDS Research (CeSHHAR), Harare, Zimbabwe; 2 Department of International Public Health, Liverpool School of Tropical Medicine, Liverpool, United Kingdom; London School of Hygiene and Tropical Medicine, UNITED KINGDOM OF GREAT BRITAIN AND NORTHERN IRELAND

## Abstract

Self-help groups (SHGs) have been effective in improving the health and wellbeing of women generally but there is little evidence on whether and how they improve HIV and sexual and reproductive health (SRH) outcomes among female sex workers (FSWs), particularly in sub-Saharan Africa. This scoping review attempted to address this gap by identifying and analysing literature on SHGs for FSWs in sub-Saharan Africa. The review followed the 5-step framework developed by Arksey and O’Malley: 1) defining the research question, 2) identifying relevant studies, 3) selecting studies, 4) charting the data, and 5) collating, summarising, and reporting the results. We searched three databases (CINAHL, Medline and Global Health) for peer-reviewed articles published between 1 January 2000 and 30 September 2024. We identified eleven studies: two were quantitative, seven were qualitative and two were mixed methods. Studies were from seven countries in sub-Saharan Africa. The studies suggested that SHGs can improve SRH outcomes and reduce HIV vulnerabilities among FSWs by providing emotional and financial support, health education, linkage to care and social capital (i.e., benefits derived from association). The studies also highlighted the need for tailored interventions that address the unique needs and challenges faced by FSWs. This scoping review highlights the crucial contribution that SHGs make in promoting social cohesion, SRH and HIV outcomes among FSWs across seven countries in sub-Saharan Africa. To build resilience and facilitate better health outcomes, FSWs need to be empowered at individual, societal and resource levels through SHGs. Further research on the formation, structure, leadership, sustainability of SHGs and contextual factors, is required for understanding the best practices of their implementation to achieve long-term success.

## Introduction

Globally, female sex workers (FSWs) are 30 times more likely to acquire HIV than general population women [1]. High numbers of sexual partners, inability to control prevention measures [2,3], stigma that fuels violence and, criminalisation of sex work, combine to reduce FSWs’ exposure to services and support, which in turn, increases their vulnerabilities to HIV and other sexually transmitted infections (STIs) [4,5]. A systematic review of community empowerment and involvement of FSWs in targeted sexual and reproductive health (SRH) interventions in Africa found that FSWs aged 15–49 years were at a higher risk of SRH morbidity, violence and discrimination compared to same age general population women [6]. However, the overall impact of empowerment interventions on FSWs was not clearly established in this review.

In sub-Saharan Africa (SSA), the epicentre of the HIV epidemic [7] the burden of HIV and other STIs among FSWs is disproportionately high [8]. For example, in Zimbabwe, HIV prevalence was estimated to be 48% among FSWs in 2022 compared to 11% among the general population of adult women [9]. It is important to note that sex work has been a contributing factor in the spread of HIV in SSA [10].

FSWs’ vulnerabilities complicate their ability to prevent or manage HIV; therefore, interventions that address these vulnerabilities are needed. Self-help groups (SHGs), where individuals with commonalities come together to support each other, have potential for impact [11]. The concept of the SHG as a catalyst for change came about in the US in the 1930s when these groups were used to help alcoholics recover [12]. The approach became widely accepted for non-alcohol addiction problems after World War II [13]. In the 1960s, civil rights movements began to evolve in many developed countries, as people became aware of their collective power. This concept emphasised high levels of group ownership, control and management concerning goals, processes and outcomes [14].

In SSA, SHGs expand upon traditional models of collective labour and savings, such as rotating savings and credit associations, which were common well before donor-led programs [15]. The first formal savings group initiative was started in Niger in the early 1990s by an international organisation [16,17]. Since then, a number of Non-Governmental Organisations (NGOs) have facilitated savings group programmes throughout the continent [18]. According to Brody et al [19], collective or individual empowerment can happen when individuals join together to address their challenges. Past research has shown that SHGs, along with other community mobilisation and structural interventions, can empower FSWs to address their economic, social, psychological and political vulnerabilities [20]. A study in India highlighted the impact of SHGs among sex workers, when FSWs who attended SHGs demonstrated higher HIV knowledge, accessed services more frequently, and were more likely to turn away clients who refused to use condoms, compared to those that did not attend SHGs [21]. However, there is a lack of substantive evidence on how SHGs can address health-related outcomes of FSWs in the SSA context. This scoping review sought to explore whether and how SHGs improve FSWs’ SRH and HIV outcomes in SSA.

## Methods

### Study design

The main objective of the scoping review was to explore a body of literature to identify what is known about SHGs in relation to addressing FSWs’ SRH and HIV outcomes in SSA. We conducted a preliminary search of MEDLINE, the Cochrane Database of Systematic Reviews and *JBI Evidence Synthesis*, and we did not identify any current or planned systematic or scoping reviews focusing on this topic. The scoping review was performed per Preferred Reporting Items for Systematic Reviews and Meta-Analyses extension for Scoping Reviews (PRISMA-ScR) guidelines (S2 Table). The scoping review started with the development of a study protocol. The study protocol was uploaded on Open Science Framework (OSF) and subsequently published [22].

We employed the 5-step framework developed by Arksey and O’Malley [23], which Levac et al. [24] and Colquhoun et al. [25] expanded on, and Peters [26] further outlined in the Joanna Briggs Institute Manual (2020 version). The five steps are: 1) defining the research question, 2) identifying relevant studies, 3) selecting studies, 4) charting the data, and 5) collating, summarising, and reporting the results [23].

### Step 1: Defining the research question

We used the Population–Concept–Context (PCC) framework [27] to identify the main concepts in the primary review question. We also used the PCC framework to develop the research objective(s) and question(s) to inform inclusion and exclusion criteria and consequently, the literature search strategy.

The primary review question was, *‘How do self-help groups influence SRH and HIV outcomes among female sex workers in SSA?*’. A sub-question to this was *‘How effective are SHGs in influencing positive SRH and HIV outcomes?* and another sub-question was ‘*What are the mechanisms through which SHGs influence these outcomes?’*. These questions enabled us to map the range of relevant literature around these aspects and inform the direction of future research. Components of the PCC framework are described below.

#### Population.

We included FSWs in SSA. The review considered FSWs as women who received money and/or goods or favours in exchange for sex. Transactional sex relationships were also considered “sex work”, even if participants did not self-identify as sex workers. We restricted the review to studies conducted within SSA between 1 January 2000 and 30 September 2024. This period was chosen because it allowed the search to include relevant articles within the SSA context considering SHGs are relatively new within this setting compared to others such as South Asia, where they have a longer and institutionalised history [28]. This period also considered that there was no similar scoping review during that time.

#### Concept.

The scoping review explored the concepts of SHGs and FSWs’ SRH and HIV outcomes. For the purposes of this review, we considered a SHG as a group of individuals with commonalities (i.e., engaged in sex work) coming together to support each other. We considered that the SHG needed to have the following elements: a common goal, voluntary membership, regular meetings, peer support and capacity-building initiatives.

#### Context.

Our review focused on sub-Saharan Africa, the area and regions of the continent of Africa that lie south of the Sahara, including Central, East, South and West Africa [29].

### Step 2: Identifying relevant studies

We identified studies relevant to this review through searching electronic databases of published literature in Medline, Global Health and CINAHL databases. The general search strategy is outlined in Table 1.

**Table 1:** Search strategy.

Concept	Search terms
Empowerment groups	“Self help group*” OR “self-help group*” OR SHG* OR “collective*” OR “empowerment group*” OR “community group*” OR “group based activit*” OR “savings group*” OR “support group*” OR “mukando” OR “psycho social support group*” OR “peer support group*” OR “support system*” OR “safety net*” OR “morale booster*”
Sexual and reproductive health	“Sexual and reproductive health” OR “SRH” OR “reproductive health” OR “health outcome*” OR “sexual health” OR “health access” OR “access to health” OR “clinic uptake” OR “unintended pregnanc*” OR “STI*” OR “gender based violence*” OR “GBV” OR “human papilloma virus” OR “HPV” OR “safe sex” OR “family planning” OR “cervical cancer” OR “safe abortion”
HIV	“HIV” OR “hiv-1*” OR “hiv-2*” OR “hiv1” OR “hiv2” OR “HIV infect*” OR “human immunodeficiency virus” OR “human immuno-deficiency virus” OR “human immune-deficiency virus” OR “acquired immunodeficiency syndrome” OR “acquired immuno-deficiency syndrome” OR “acquired immune-deficiency syndrome” OR “HIV Infection*”
Female sex worker	“Female sex worker*” OR “FSW” OR “sex worker*” OR “prostitute*” OR “thigh vendor*” OR “transactional sex*” OR “bar maid*” OR “sex work*” OR “girls selling sex” OR “women selling sex” OR “female* selling sex” OR “young women selling sex” OR “transact* sex” OR “exchang* sex” OR “sell* sex” OR “sold sex” OR “trad* sex” OR “commercial sex” OR “escort” OR “hooker*” OR “streetwalker*” OR “whore” OR “hustler*” OR “woman of the street*” OR “bawd” OR “call girl*” OR “courtesan” OR “drab*” OR “tart*” OR “harlot*” OR “slut*”
Sub-Saharan Africa	“Africa, south of the Sahara” OR “sub-Saharan Africa” OR “Angola” OR “Benin” OR “Botswana” OR “Burkina Faso” OR “Burundi” OR “Cameroon” OR “Cape Verde” OR “Central African Republic” OR “CHAD” OR “Comoros” OR “Congo” OR “Congo Democratic Republic” OR “Djibouti” OR “Equatorial Guinea” OR “Eritrea” OR “Ethiopia” OR “Gabon” OR “Gambia” OR “Ghana” OR “Guinea” OR “Guinea-Bissau” OR “Cote d’Ivoire” OR “Ivory Coast” OR “Kenya” OR “Lesotho” OR “Liberia” OR “Madagascar” OR “Malawi” OR “Mali” OR “Mozambique” OR “Namibia” OR “Niger” OR “Nigeria” OR “Sao tome and Principe” OR “Rwanda” OR “Senegal” OR “Seychelles” OR “Sierra Leone” OR “Somalia” OR “South Africa” OR “South Sudan” OR “Sudan” OR “Swaziland” OR “Tanzania” OR “Togo” OR “Uganda” OR “Zambia” OR “Zimbabwe”

### Step 3: Selecting studies

On 20^th^ April 2024, the lead author (GM) tested a general search strategy by running it using a Boolean search on the Discover platform, combining the search terms from the PCC (Population, Concept Context) framework using “AND” and separating different terms using “OR”. She then searched each of three databases (Medline, Global Health and CINAHL) using the key terms from the PCC framework to find the Medical Subject Headings (MeSH) used in the databases. She then combined the MeSH terms with the free text in the search strategy that she developed (S1 Table). These databases were carefully selected for their comprehensiveness in covering the area under research. The researchers’ familiarity with the databases also enhanced efficiency in the search process. Search terms were determined with input from the research team, research collaborators and knowledge users. Searches were limited to literature published in English only and literature published before 2000 was excluded so that the review considered the most recent publications. Search results were downloaded and imported into Endnote 20. After removing duplicates in Endnote 20, the articles were exported to Covidence, a collaborative software. The search was closed on 30^th^ September 2024.

Once the articles were imported into Covidence, duplicates were removed again. The review process then started. It consisted of two levels of screening: (1) a title and abstract review and (2) full-text review in parallel. GM and a co-reviewer (ON) did the first and second level of screening. Any conflicts involved a third reviewer (WM).

#### Inclusion and exclusion criteria.

Table 2 shows the review’s eligibility criteria, including the rationale.

**Table 2:** Studies eligibility criteria.

Criteria	Inclusion criteria and rationale	Exclusion criteria and rationale
Population	- Articles reporting on FSWs, even if they focused on other population groups. We focused on FSWs as they are disproportionately affected by HIV in SSA [8].	- If articles did not report on FSWs at all.
Concept	- Articles reporting on SHGs (with all or any of the following elements: voluntary membership, regular meetings, peer support, and capacity-building initiatives). - Articles reporting on SRH and HIV.	- Articles that reported on any grouping of FSWs, without any of the elements outlined in the working definition. - Articles not in any way related to SRH and HIV.
Context	- Studies conducted in any country within SSA.	- Studies conducted outside SSA.
Study design/type of evidence	- Articles should have been peer-reviewed and published between 1st January 2000 and 30^th^ September 2024. - Articles reporting findings from primary research. - Articles published in English.	- Grey literature, unpublished sources. - Articles published before 1st January 2000. - Reviews (e.g., systematic or scoping. reviews) - Non-English articles as we did not have the resources to analyse articles published in other languages.

Articles that had a title and/or abstract that seemed eligible were included in this first level of screening. These articles reported on FSWs and had some aspect of participation in SHGs within the SSA context.

The second stage of the review involved GM and ON reading through all the articles that were included for the full-text review. To determine inter-rater agreement for articles to include in the full text review, Covidence software was used. Any discordant full-text articles were reviewed a second time and further disagreements about study eligibility at the full-text review stage were resolved through discussion with a more senior researcher (WM) until full consensus was obtained.

### Step 4: Charting the data

Data charting is the process of data synthesis and interpretation by categorising, visualising and structuring information regarding key themes and issues [24]. Following Levac et al.’s guidance, we developed a standardised Excel template for data charting. The template included essential study information: author(s), year of publication, title of the study, geographical setting, study design, methodologies applied, and key themes related to our review objectives. The process of extraction was iterative in nature; we pulled data in waves, refining the template in each wave.

We began the data charting process with a pilot phase where two independent reviewers (GM and ON) charted data from a random sample of 5–10 of the final selected studies. This piloting process determined whether or not both reviewers’ independent chartings aligned with the review objectives, allowing for any changes to the data charting form. Any disagreements were discussed with a senior researcher (WM), and the data charting form was revised accordingly.

### Step 5: Collating, summarising, and reporting the results

We used descriptive statistics to present included studies’ characteristics. Specifically, we presented the number of studies meeting study criteria (e.g., study design type [qualitative, mixed methods or quantitative], geographical location).

Following best practices in thematic analysis, which include being purposefully iterative and reflexive, we then thematically examined the main conclusions drawn from the included studies [30]. Before logically developing and applying preliminary codes and themes to the data retrieved from the chosen studies, GM and ON first carefully reviewed and comprehended the extracted data. We then developed an initial codebook.

In order to continuously improve the thematic analysis process, we discussed and shared notes throughout. For instance, we were able to create new codes and themes or combine existing ones when there were overlaps. In order to better comprehend our findings and pinpoint pertinent gaps in relation to our review questions, we finally merged all of our studies and mapped out the similarities and differences in our data [31]. This final analysis was developed and refined with input from all co-authors.

### Ethics and dissemination

Ethical approval was not required as this was a literature review.

## Results

### Study descriptions

**Fig 1:**
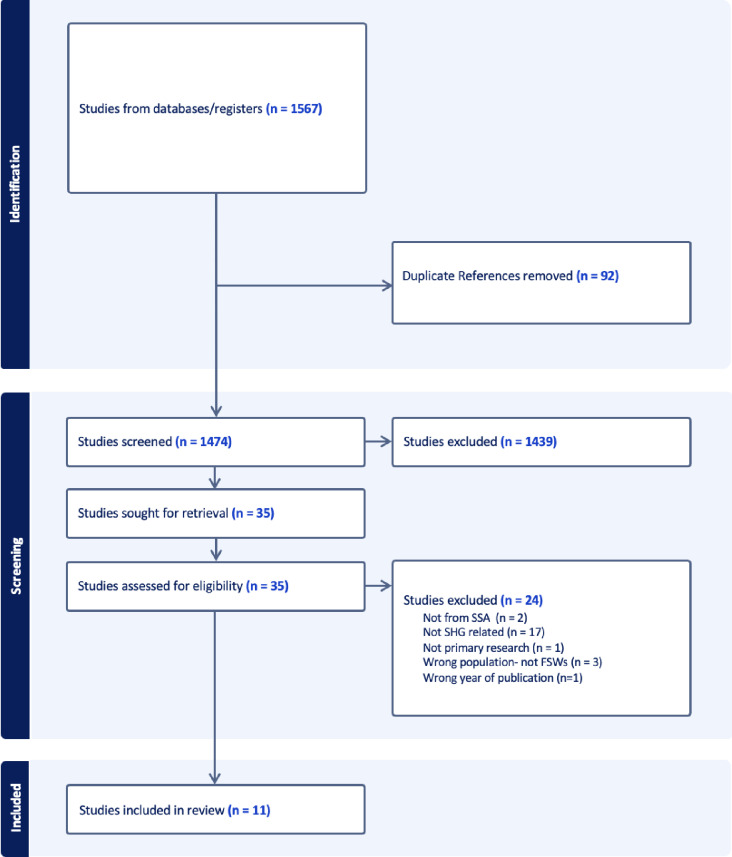
PRISMA flowchart of study selection process.

The search yielded 1,567 potentially relevant records from the three databases; 92 records were identified by Covidence to be duplicates and 1,474 records remained for title and abstract screening. Title and abstract screening were done by two reviewers and there were 36 conflicts from the records (i.e., reviewers disagreed). The conflicts were mainly on articles that were not SHG-related but seemed to look at issues to do with empowerment. The review team resolved conflicts by going through the titles and abstracts together. The studies were excluded as they did not have the requisite elements of a SHG (outlined earlier). There remained 35 articles for full text screening. After cross-validation and full text screening, 11 articles were selected. Fig 1 below presents a summary of the selection process.

### Study characteristics

The 11 studies included for final review were conducted in seven SSA countries. One study was multi-country and conducted in three countries: Kenya, Tanzania and Uganda [32]. Four of the studies were conducted in Tanzania and were of the same intervention and trial (Project Shikamana, 4.1 to 4.3) [33–36]. One study was conducted in Zimbabwe [37], another was from Cameroon [38], one from Côte d’Ivoire [39], one from South Africa [40] and two from Kenya [41,42] (Table 3).

**Table 3:** Study characteristics.

	Authorship	Country	Setting	Study Design	Sample size	Study period	Type(s) of SHG identified	Study aim/ objectives	Main findings
1	Cange, C.W., et al., 2017	Cameroon	Peri-urban	Qualitative	100	Unspecified	Njangi – a form of microfinance, with FSWs meeting regularly in groups to support each other and to create cohesion	To qualitatively investigate the impact of social cohesion, as well as individual and community resilience strategies, on sex work empowerment in Cameroon.	FSWs reported that they were faced with challenges of physical violence, sexual assault and demands for bribes to avoid fines and or imprisonment from authorities. They described strategies such as “looking out for each other” when faced with security threats.
2	Chingono, R., et al., 2022	Zimbabwe	Peri-urban	Mixed methods	93	8 months (February - September 2019)	ISALS	This study aimed to explore whether and how participation in a SHG intervention affected the experiences and perceptions of mental health stressors of vulnerable young mothers’ who sell sex.	This study found that building support, reducing isolation and providing practical and material assistance helped meet many of adolescent girls and young women (AGYW)’s (who were also FSWs) needs related to their struggles to cope with looking after their children in a context of severe economic deprivation. The expansion of social networks seemed to benefit participants and this seemed to reduce stress and anxiety through meeting and discussing shared experiences with peers.
3	Huschke, S., 2019	South Africa	Urban	Qualitative	32	December 2015 and July 2017	Creative Space workshops (CSW)- risk reduction workshops	To investigate the social and psychological effects of peer-led risk-reduction workshops for sex workers in Soweto, South Africa, with a particular focus on the ways in which they might contribute to community empowerment.	CSW offered an environment for FSWs to come and vent out and share without fear of being judged. However, there was the feeling among interviewees that there were knowledge sharing limitations in a sex worker-only space. They suggested that medical and legal experts should be invited more regularly to the workshops. Sex workers also suggested that they should receive more training to be able to respond medical and legal questions.
4.1	Kerrigan, D., et al., 2019	Tanzania	Fishing beach	Quantitative (RCT)	496	October 2015 to April 2016	Informal networks and savings groups	To determine the impact of a community empowerment model of combination HIV prevention (Project Shikamana) among female sex workers (FSW) in Iringa, Tanzania on HIV incidence.	Project Shikamana suggested that a community empowerment-based approach to HIV prevention was both feasible and highly effective in sub-Saharan Africa. It highlighted a reduction of HIV incidence because of the intervention. This study suggested that community empowerment based models of HIV prevention be expanded.
4.2	Leddy, A.M., et al., 2020	Tanzania	Rural	Qualitative	36	August 2016- August 2018	Informal networks and savings groups	This analysis aimed to describe the community empowerment process among female sex workers in Iringa, Tanzania, in the context of a randomised controlled trial of a community empowerment-based model of combination HIV prevention.	Participants viewed the Drop-in-Centre as a place where they could openly be ‘working women’ (the term they used to refer to themselves), share their stories and concerns, and be treated with respect and dignity, which was not how they were typically treated in other spaces.
4.3	Mantsios, A., et al., 2018	Tanzania	Farming area	Quantitative	496	Not specified	Community savings groups	This analysis aimed to assess the association between participating in a community savings group and consistent condom use (CCU) among FSW in Iringa, Tanzania.	Study findings indicated that community savings groups may play an important role in reducing sexual risk behaviours of FSWs and hold promise as part of comprehensive, community-led HIV prevention strategies among FSWs. Study found that individuals who were participating in savings groups had a higher chance of CCU with new clients in the last 30 days than those who were not part of a savings group.
4.4	Mantsios, A., et al., 2018	Tanzania	Urban	Qualitative	60	April 2015 to February 2016	Community savings group *(michezo*)	This paper describes the formative work for Project Shikamana. It aimed to examine the potential for community savings groups (locally called *michezo*) among FSW in Iringa, Tanzania to reduce HIV risk and promote economic and community empowerment.	Women who participated in this pilot project of the Shikamana intervention felt that *michezo* provided them with financial and social support. They felt a sense of solidarity and collectivism from participating in *michezo.* Group participation influenced women’s reported HIV risk. Some of the tensions and challenges experienced within the groups included a perceived sense of exclusion of certain women from participating. Women also expressed a desire to have the groups formally registered and become recognised by the broader community.
5	Nakamanya, S., et al., 2022	Kenya, Tanzania and Uganda	Urban	Qualitative	96	March 2018 to June 2019	Social network groupings (exchange of information)	The study aimed to understand the different social networks among women working in fishing communities of Lake Victoria and also understand their interaction with women’s mobility patterns and HIV risk.	Women’s networks helped them find employment as well as social security whereby they saved, donated and lent money to one another through their associations known as ‘*Ifogong’o’* in Sukuma language (meaning savings pouch/purse [in Tanzania-TZ] and ‘*Munno mukabi’* in Luganda language (meaning ‘a friend in need’ self-help group [in Uganda-UG]).
6	Namey, E., et al., 2018	Cote d I’voire	Urban	Mixed methods approach	78	September - October 2014	Savings groups	The study aimed to better inform economic strengthening programs for HIV risk reduction with FSWs.	Group savings meetings were conducted regularly and members of the group were self-selected based on already existing relationships. This facilitated the introduction or reinforcement of health promotion and/or risk reduction messages.
7	Nathenson, P., et al., 2017	Kenya	Urban and rural	Qualitative	29	November 2012 for 6 months	Women’s cooperative, empowerment in business…fishing	The study aimed to describe a case study of an intervention in Kenya that empowered female fishmongers with business skills, boat ownership and increased income so that they no longer had to trade sex for fish.	Participants were able to build three boats and women became profitable boat managers. Women were empowered through ownership and both men and women received skills training which empowered them as business people.
8	Wanjiru, R., et al., 2022	Kenya	Urban	Qualitative	40	October 2019	Sex Workers Outreach Program (SWOP) clinics- support systems that developed because of the SWOP, friendships among clinic attendees	To explore how female sex workers in Nairobi, Kenya, utilise different resources to navigate the negative consequences of the work they do.	Sex workers reported that they benefited from a support system with other sex workers that developed while using the safe spaces within the SWOP Clinics. Over time. friendships grew amongst the clinic attendees, leading to strategies being devised to encourage and help each other. Encouraging FSWs to come together and advocate together for their needs was a key resource from which resilience and forbearance could grow. This would increase their negotiating power as a resilience asset, minimising exposure and risk.

While the review primarily focused on FSWs, two of the articles were diverse and FSWs were studied within the context of other populations such as fishermen, HIV care providers, police, venue managers, community advisory board members and research staff [32,43] (Table 3).

**Fig 2:**
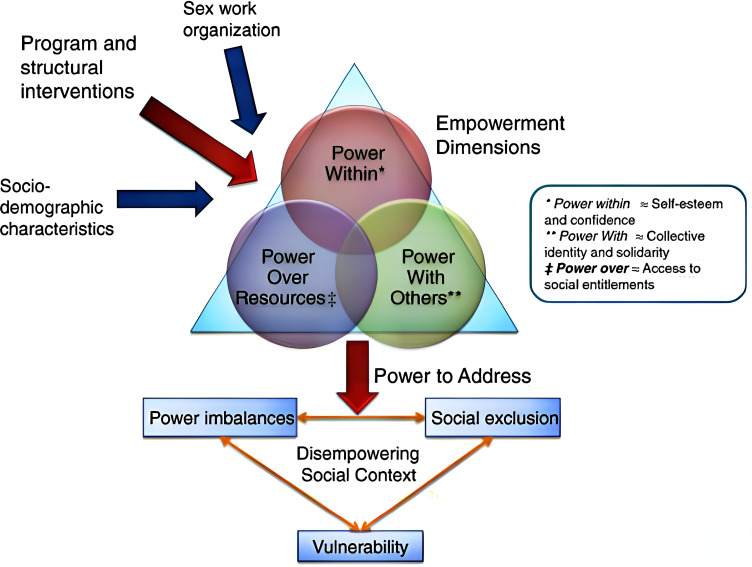
Integrated empowerment framework. Source: Blanchard et al. BMC Public Health 2013, 13:234.

Identified major themes mapped well onto the integrated empowerment framework for FSWs which combines various elements and strategies aimed at empowering individuals or communities. The three main domains of this framework are power within, power with (others), and power over (resources) [44–46]. The framework is depicted in Fig 2 below. We also integrated other emergent themes beyond this framework, adding depth to our understanding of how SHGs relate to HIV and SRH outcomes among FSWs.

### Power within

The concept of “power within” refers to the extent of influence, control and decision-making authority that individuals or groups possess within their immediate social and environmental contexts [47]. It relates to the degree to which an individual or group can exercise their agency and affect their own lives or the lives of others within their microsystem [47].

Being a member of a SHG empowered FSWs with knowledge of HIV prevention services. For example, in the Kenyan study, most of the participants mentioned having little or no knowledge of HIV prevention before engaging with the Sex Workers Outreach Program (SWOP) clinic services [39,41]. However, FSWs who took part in the study reported that after receiving the services and information provided by SWOP’s “sex worker-friendly” clinics, they no longer agreed to engage in sex without a condom. This change in thinking was based on their realisation of the purely monetary nature of their relationships with clients [41].

In the SWOP clinics, some participants also reported using pre-exposure prophylaxis because they were now more aware of the risks involved, and should there be a condom failure, they were protected [39]. The empowerment they gained by being part of a SHG helped FSWs recognise their self-worth and choose whom to have sex with and reduced their reliance on men [39]. This Kenyan study suggests that other health outcomes, such as modern family planning use, may also be influenced by these interventions, especially when family planning is part of a comprehensive empowerment-based programme [41].

Two studies found statistically significant associations between higher pay per sexual encounter, higher total income, higher sex work income and group participation [33,35]. Community savings group participation was significantly associated with reported consistent condom use (CCU) with new clients and regular clients (but not with steady, non-paying partners) [35]. Higher income was associated with CCU with a new partner [35]. CCU with a regular partner was also associated with higher income and other factors such as savings group participation, older age, longer time in sex work, and having financial dependents. Overall, SHGs enhanced FSWs’ agency, which aligns with the “power within” concept.

### Power with others

“Power with others” denotes a collaborative and shared form of authority where individuals or groups come together in pursuit of common objectives or collectively address issues [47]. It embodies the concept that power is not concentrated within a single entity or individual; instead, it is distributed among the participants in a cooperative manner [47]. Within this framework, individuals or groups join forces, combining their collective resources, knowledge and influence so that they effect change, make decisions, or achieve tasks, usually to aid fairness, social justice or mutual benefit [47].

Most of the FSWs reported benefiting from a support system with other sex workers, their local community, that they had devised to encourage and help each other out [37,41]. This support included, but was not limited to: financial and emotional support, sharing of health education information, and linkage to health care services [41]. Others also highlighted how they helped each other to seek medical assistance during illness and, in some cases, even accompany each other to the health facility for treatment [36,37,48]. Where they faced violence from clients, they assisted each other through telephone-based security strategies where they called each other for assistance [48]. Additionally, they offered each other guidance on issues such as condom breakage, partner violence and matters related to child support and care. It is also within these SHGs that they advised each other on the dangers of drug and alcohol use. These bonds and associations within their peer groups provided solace and the means to thrive, despite numerous challenges [41].

Social capital (i.e., benefits derived from association) was a resource for resilience which held great value among FSWs. Furthermore, the act of coming together allowed FSWs to address instances of abuse by security forces during their work [48]. FSWs also trained each other on how to survive in the industry and on social security. Also, they taught each other about condom use [32,33,36].

Creative Space Workshops offered FSWs a platform to address common shared vulnerabilities. FSWs recognised the therapeutic value of peer-led group sessions, where they shared their personal struggles and traumas. These sessions contributed to enhancing sex workers’ self-esteem by fostering a supportive, non-judgmental, rights-based discourse, which led to a reframing of sex work as legitimate employment, and portrayed sex workers as individuals with inherent rights [40]. They also exposed sex workers to valuable health knowledge, broadened their understanding of their legal rights, and informed them on available support services. In this way, Creative Space Workshops promoted a greater sense of emotional well-being, and was experienced as empowering, instilling a greater sense of agency in the face of pervasive structural and interpersonal challenges. It also fostered hope that their circumstances could improve. Overall, the group concept and collective empowerment illustrated the “power with others” concept.

### Power over resources

“Power over resources” pertains to the capacity of individuals, organisations or entities to exercise control, distribute and make decisions regarding assets, commodities or properties [49]. This power capacitates them to determine how these resources are used, distributed and managed [49]. It is an essential aspect of resource management, and can have significant implications for individuals and communities and how they manage SRH and vulnerability to HIV [49].

In the context of savings funds, that is, where FSWs jointly contribute some money and oversee the disbursement of funds within SHGs, they were generally perceived as empowering [48], and empowerment had a positive influence on sexual risk behaviour. Although some SHGs gave FSWs power over resources, they fell short of establishing a framework for FSWs to secure and manage long-term savings. These savings would typically be earmarked for purposes such as transitioning from sex work, home ownership, child support, marriage, accessing essential healthcare or returning to their places of origin [50]. However, one study clearly demonstrated the “power over resources” when FSWs were able to register a catering business [34]. They formalised their activities by registering with the government as an official business which grew, registering membership of 50 FSWs, and became self-sustained and continued meeting even after the study [34]. However, it was not established in this study how far this empowerment impacted on SRH and HIV outcomes.

## Discussion

The benefits derived from participating in SHGs for FSWs predominantly revolve around the empowerment of the individual (power within) and the strength they gain from uniting as a group (power with others). However, in most of the articles reviewed, achieving “power over resources” remains a challenge. Empowerment strategies have been employed to increase FSWs’ access to social entitlements, financial credit and educational opportunities, all of which open up more choices for them in terms of decisions that impact their HIV and SRH outcomes.

In India, for example, significant progress has been made with regards to achieving “power over resources” but this is something that takes time [51]. One good example is the Usha Multi-purpose Cooperative Society Limited (USHA), which is the largest and first ever sex worker-led financial institution in Southern Asia [51]. USHA was formed in 1995 and it is now one of the biggest financial institutions run by sex workers. One of the factors that influenced sex workers to start this initiative was the need for financial freedom in face of various vulnerabilities including stigma and discrimination. This drive facilitated the group’s survival.

Time, among other variables, was an important factor, highlighting that for “power over resources” to be achieved, a significant amount of time and consistency in the group is relevant and necessary. This time factor could not be fully explored in this review because of the short-term nature of the studies. Achieving “power over resources” requires sustained effort and consistency within the group. Most of the included studies were of short duration, with most lasting less than one year. This relatively short intervention period may have limited the ability to observe the long-term results that are anticipated from participation in SHGs, including improved mental and physical health, reduced risk of HIV acquisition, and decreased HIV transmission, among other SRH concerns. Therefore, some of these desired long-term outcomes may not have been apparent due to the brief nature of the studies.

The organisation and structure of the group, group activities and leadership were some of the other factors that influenced member empowerment, but were not fully investigated in this analysis. A study on SHGs in Hong Kong highlighted the interrelationships among SHG participation, social support, social learning, leadership and empowerment [52]. It emphasised that effective leadership within SHGs can inspire growth and foster an environment conducive to empowerment. Study findings indicated that members experienced enhanced intrapersonal, interpersonal and community/political empowerment as a result of their participation in well-structured groups with strong leadership [52].

This review has underlined that, at both the individual and societal levels, empowerment is key to FSWs’ effective response to their health vulnerabilities. Empowerment can only be achieved by addressing the structural issues which in the first instance create these vulnerabilities. As noted in the literature [51,52], comprehensive change is essential; community mobilisation strategies for FSWs must be complemented by structural interventions that address the underlying social, economic, legal and political factors contributing to their disempowerment. Such a multifaceted approach is necessary to create sustainable pathways towards empowerment and improved well-being for FSWs.

While numerous studies have underscored the manifold benefits of SHG participation, it is essential to recognise that they do not create an idealised, conflict-free environment where the challenges sex workers face in their daily lives cease to exist. One prominent issue highlighted in the literature pertains to the tensions that can arise between different groups of sex workers and between sex workers and non-sex workers who occasionally engage in these groups to access associated benefits [32,40]. Trust emerges as another crucial factor that significantly influences the success of SHGs. In some studies, trust issues were evident as some FSWs expressed reservations about trusting their peers which, in turn, had repercussions for SHGs’ overall effectiveness.

Evidence from the included studies demonstrates that the domains of power: “power within”, “power with others” and “power over resources”, are intricately woven into the empowerment process, which is a necessary enabling factor for SHGs to effectively influence HIV and SRH outcomes. However, it must be underlined that empowerment alone is not the end; rather, the operational functionality and sustainability of SHGs themselves depend on a number of other factors, including group formation, leadership dynamics, the approaches employed by SHGs, and the broader context in which they find themselves. These elements will decide how well SHGs perform in terms of achieving the intended health-related results.

Recognising the importance of these additional factors, this literature review found minimal data specifically addressing them, and it thus limited the ability of the authors to comprehensively include these in their analysis. Hence, the main focus has been on the three domains of power, as this was most strongly supported by the literature. This focus enables us to underline mechanisms through which empowerment operates within SHGs, while recognising that a more holistic understanding of SHG dynamics would require further investigation into these other critical elements. Future research should be conducted for in-depth investigation of these dimensions to further enhance the effectiveness of SHGs in bringing about improved health outcomes among FSWs, including making the interventions responsive to diverse needs and challenges faced by the group.

## Recommendations for policy and practice

1. Long-term commitment: Most of the studies included in this review were short-term, limiting our ability to assess comprehensive effects that SHGs may have on the health and well-being of FSWs. Further research and interventions need to focus on longer-term engagement to track sustained improvement in mental and physical health, reductions in HIV acquisition, and decreases in onward transmission of HIV and other SRH issues.
2. Sustainability of SHGs: SHGs should be organised and maintained in a manner that ensures sustainability of the groups so that benefits gained from these could be sustained sufficiently long for appreciable and measurable improvements to result in health and well-being for the FSWs.
3. Conflict resolution and trust-building: SHGs are not exempt from the various conflicts and tensions that FSWs experience in everyday life. Conflicts within SHGs would have to be resolved to further enhance the scope of these groups. Enhancing trust-building activities and promoting the importance of trust within the groups likely enhances their success.
4. Diverse group dynamics: Recognise the diversity of background, experiences and needs among FSWs in the SHGs. Interventions may therefore be usefully targeted at subgroups within this heterogeneous population to deal effectively with subgroup-specific issues and vulnerabilities.
5. Structural and policy support: Although SHGs may empower FSWs at both the individual and collective levels, such a community-based initiative needs to be supported by structural changes. This calls for advocacy for social, economic, legal and political support, including increased access to social entitlements, financial credit and educational opportunities.
6. Holistic approach to health: SHGs should be integrated into a comprehensive SRH and HIV prevention framework that comprises information and services on HIV prevention, access to healthcare, family planning and substance use support.
7. Community mobilisation: The participation of FSWs in SHGs should be part of broader community mobilisation efforts. That FSWs have been actively involved in addressing health vulnerabilities should be complemented by changes in social and cultural norms that contribute to stigma and discrimination.

## Strengths and limitations

This scoping review allowed us to broadly examine the different types of SHGs existing in SSA and assess how they influence SRH and HIV among FSWs. The focus on urban, rural, peri-urban and fishing communities enhances the richness of findings despite not being generalisable because of the qualitative nature of the research. A limitation in this scoping review was the exclusion of grey literature, where most of practice evidence and insights into SHGs can be obtained, which may not be captured through peer-reviewed academic material. Focusing on peer-reviewed literature only may have excluded critical viewpoints of NGOs, community-based organisations and practitioners who work directly with SHGs.

Another limitation is that we used only three search engines–Medline, Global Health and CINAHL–to conduct the article search despite knowing that other search engines may have yielded studies not included here. This was because conducting a scoping review involves substantial time and resources and inherent constraints led us to focus on the three well-curated and widely used databases. We carefully selected these three databases to minimise redundancy in our search results, considering the potential for overlapping content across multiple databases. They are comprehensive and they maximised the depth and relevance of the literature that was retrieved. This contributed positively to the overall quality of the review. Another limitation relates to the relatively few studies found, mostly published recently because SHGs are an emerging area of research in Africa, that needs to be expanded. Finally, we only included studies published in English which may have introduced language bias.

## Conclusions

SHGs have shown great promise in empowering FSWs through promotion of social cohesion, SRH and HIV outcomes across seven SSA countries. FSWs are more resilient and have better health outcomes when they are empowered at individual, societal and resource levels. To fully harness the potential of SHGs, it is crucial to adopt a holistic, long-term and community-centred approach, address trust and conflict issues and advocate for broader structural changes that support FSWs’ well-being and rights. By doing so, we can contribute to a more comprehensive and effective response to the complex health challenges faced by this vulnerable population.

## Supporting information

S1 Table Mesh and free text search strategy. (DOCX)

S2 Table Preferred Reporting Items for Systematic reviews and Meta-Analyses extension for Scoping Reviews (PRISMA-ScR) Checklist. (DOCX)
