# Supplementary material for: Exploring the contribution of self-help groups to sexual and reproductive health and HIV outcomes for female sex workers in sub-Saharan Africa: A scoping review
Source: PLOS Glob Public Health. 2025 Apr 24;5(4):e0003883. doi: 10.1371/journal.pgph.0003883 (PMC12021211; doi:10.1371/journal.pgph.0003883)
Supplement: S1 Table — (DOCX) [file pgph.0003883.s001.docx]

S1 Table: Mesh and free text search strategy

| **Friday 12 April 2024** | | | | |
| --- | --- | --- | --- | --- |
| **#** | **Query** | **Limiters/Expanders** | **Last Run Via** | **Results** |
| S1 | MESH TERMS  (MH "Empowerment") OR (MH "Social Skills") OR (MH "Social Conformity") OR (MH "Social Dominance") OR (MH "Social Inclusion") OR (MH "Help-Seeking Behavior")  FREE TEXT  “Self help group*” OR “self-help group*” OR SHG* OR “collective*” OR “empowerment group*” OR “community group*” OR “group based activit*” OR “savings group*” OR “support group*” OR “mukando” OR “psycho social support group*” OR “peer support group*” OR “support system*” OR “safety net*” OR “morale booster*” | **Limiters** - Full Text; Publication Date: 20000101-20241007; Abstract Available; English Language; Human; Citation Subset: MEDLINE; Language: English; Peer Reviewed  **Expanders** - Apply equivalent subjects  **Search modes** - Boolean/Phrase | EBSCO Host | **190,687** |
| S2 | MESH TERMS  (MH "Sexual Health") OR (MH "Reproductive Health") OR (MH "Reproductive Health Services") OR (MH "Reproductive Rights") OR (MH "Sexual Behavior+") OR (MH "Sexual Partners") OR (MH "Reproductive Behavior+") OR (MH "Sexually Transmitted Diseases+") OR (MH "Health") OR (MH "Health Status") OR (MH "Sexually Transmitted Diseases, Bacterial") OR (MH "Health Behavior+") OR (MH "Health Promotion+") OR (MH "Sexually Transmitted Diseases, Viral+") OR (MH "Women's Health+") OR (MH "Occupational Health") OR (MH "Adolescent Health") OR (MH "Health Literacy+") OR (MH "Mental Health") OR (MH "Health Equity") OR (MH "Maternal Health") OR (MH "Women's Health Services+") OR (MH "Population Health+") OR (MH "Attitude to Health+") OR (MH "Social Determinants of Health")  FREE TEXT  “Sexual and reproductive health” OR “SRH” OR “reproductive health” OR “health outcome*” OR “sexual health” OR “health access” OR “access to health” OR “clinic uptake” OR “unintended pregnanc*” OR “STI*” OR “gender based violence*” OR “GBV” OR “human papilloma virus” OR “HPV” OR “safe sex” OR “family planning” OR “cervical cancer” OR “safe abortion” | **Limiters** - Full Text; Publication Date: 20000101-20241007; Abstract Available; English Language; Human; Citation Subset: MEDLINE; Language: English; Peer Reviewed  **Expanders** - Apply equivalent subjects  **Search modes** - Boolean/Phrase | EBSCOHOST | **2,414,472** |
| S3 | MESH TERMS  (MH "HIV+") OR (MH "HIV Infections+")  FREE TEXT  “HIV” OR “hiv-1*” OR “hiv-2*” OR “hiv1” OR “hiv2” OR “HIV infect*” OR “human immunodeficiency virus” OR “human immuno-deficiency virus” OR “human immune-deficiency virus” OR “acquired immunodeficiency syndrome” OR “acquired immuno-deficiency syndrome” OR “acquired immune-deficiency syndrome” OR “HIV Infection*” | **Limiters** - Full Text; Publication Date: 20000101-20241007; Abstract Available; English Language; Human; Citation Subset: MEDLINE; Language: English; Peer Reviewed  **Expanders** - Apply equivalent subjects  **Search modes** - Boolean/Phrase | EBSCOHOST | **230,622** |
| S4 | MESH TERMS  (MH "Sex Workers") OR (MH "Sex Attractants") OR (MH "Sex Work")  FREE TEXT  “Female sex worker*” OR "FSW” OR “sex worker*” OR “prostitute*” OR “thigh vendor*” OR “transactional sex*” OR “bar maid*” OR “sex work*” OR “girls selling sex” OR “women selling sex” OR “female* selling sex” OR “young women selling sex” OR “transact* sex” OR “exchang* sex” OR “sell* sex” OR “sold sex” OR “trad* sex” OR “commercial sex” OR "escort" OR "hooker*" OR "streetwalker*" OR "whore" OR "hustler*" OR "woman of the street*" OR "bawd" OR "call girl*" OR "courtesan" OR "drab*" OR "tart*" OR "harlot*" OR "slut*" | **Limiters** - Full Text; Publication Date: 20000101-20241007; Abstract Available; English Language; Human; Citation Subset: MEDLINE; Language: English; Peer Reviewed  **Expanders** - Apply equivalent subjects  **Search modes** - Boolean/Phrase | EBSCOHOST | **48,176** |
| S5 | FREE TEXT  “Africa, south of the Sahara” OR “sub-Saharan Africa” OR “Angola” OR “Benin” OR “Botswana” OR “Burkina Faso” OR “Burundi” OR “Cameroon” OR “Cape Verde” OR “Central African Republic” OR “CHAD” OR “Comoros” OR “Congo” OR “Congo Democratic Republic” OR “Djibouti” OR “Equatorial Guinea” OR “Eritrea” OR “Ethiopia” OR “Gabon” OR “Gambia” OR “Ghana” OR “Guinea” OR “Guinea-Bissau” OR “Cote d'Ivoire” OR “Ivory Coast” OR “Kenya” OR “Lesotho” OR “Liberia” OR “Madagascar” OR “Malawi” OR “Mali” OR “Mozambique” OR “Namibia” OR “Niger” OR “Nigeria” OR “Sao tome and Principe” OR “Rwanda” OR “Senegal” OR “Seychelles” OR “Sierra Leone” OR “Somalia” OR “South Africa” OR “South Sudan” OR “Sudan” OR “Swaziland” OR “Tanzania” OR “Togo” OR “Uganda” OR “Zambia” OR “Zimbabwe” | **Limiters** - Full Text; Publication Date: 20000101-20241007; Abstract Available; English Language; Human; Citation Subset: MEDLINE; Language: English; Peer Reviewed  **Expanders** - Apply equivalent subjects  **Search modes** - Boolean/Phrase | EBSCOhost | **383,955** |
| S6 | S1 AND S2 AND S3 AND S4 AND S5 | **Limiters** - Full Text; Publication Date: 20000101-20241007; Abstract Available; English Language; Human; Citation Subset: MEDLINE; Language: English; Peer Reviewed  **Expanders** - Apply equivalent subjects  **Search modes** - Boolean/Phrase | EBSCOhost | **75** |
